# Little Support for Discrete Item Limits in Visual Working Memory

**DOI:** 10.1177/09567976211068045

**Published:** 2022-06-17

**Authors:** Klaus Oberauer

**Affiliations:** Department of Psychology, University of Zurich

**Keywords:** working memory, continuous reproduction, capacity

## Abstract

Some theorists argue that working memory is limited to a discrete number of items and that additional items are not encoded at all. Adam et al. (2017) presented evidence supporting this hypothesis: Participants reproduced visual features of up to six items in a self-chosen order. After the third or fourth response, error distributions were indistinguishable from guessing. I present four experiments with young adults (each *N* = 24) reexamining this finding. Experiment 1 presented items slowly and sequentially. Experiment 2 presented them simultaneously but longer than in the experiments of Adam et al. Experiments 3 and 4 exactly replicated one original experiment of Adam et al. All four experiments failed to replicate the evidence for guessing-like error distributions. Modeling data from individuals revealed a mixture of some who do and others who do not produce guessing-like distributions. This heterogeneity increases the credibility of an alternative to the item-limit hypothesis: Some individuals decide to guess on hard trials even when they have weak information in memory.

Adam et al. (2017) published an influential article reporting results that they interpreted as “clear evidence for item limits in visual working memory.” They referred to the hypothesis that the capacity of visual working memory (VWM) is a discrete number of place holders (or “slots”), each of which can hold one item. Their evidence comes from a whole-report continuous-reproduction test of VWM: Participants remembered arrays of up to six colors or orientations and reported every item in that array by reproducing it on a continuous response scale, such as a color wheel. In the main experiments, participants were free to report the items in any order. Adam and colleagues found that people had a strong preference to recall the items in descending order of how well they remembered them, so that the report error—the deviation of the reproduced feature from the true target feature—increased sharply from the first to the last reported item. For items reported after the first three, participants’ responses were statistically indistinguishable from pure guessing (i.e., a uniform distribution of responses over the entire circular response scale). Adam et al. concluded that participants were able to hold only three items in VWM and had no memory information at all about any additional items. This conclusion is in line with the discrete-capacity hypothesis but contrary to alternative theories, which share the assumption that all array items are encoded into VWM to some extent (Oberauer & Lin, 2017; Schneegans & Bays, 2017; van den Berg et al., 2012).

The series of experiments I report here was motivated by the aim to identify boundary conditions for Adam et al.’s finding. I started with the assumption that by presenting arrays simultaneously and very briefly, Adam et al. might have made it difficult for participants to even encode all items, especially at higher memory set sizes. Such encoding limits could have led to states of no information in VWM about some items, even without a discrete item limit on VWM. To test that possibility, I presented the array items in Experiment 1 slowly and sequentially, and then asked participants to make a whole report of all items in the participants’ chosen order. That experiment showed no evidence for zero-information states in VWM. Experiment 2 took a step back toward the original experiments by Adam et al., presenting the arrays simultaneously but long enough for encoding every item. Again, I found no evidence for zero-information states. Experiments 3 and 4 therefore were exact replications of the materials and procedure of one of the experiments by Adam et al.; Experiment 3 tested the full range of set sizes investigated by Adam and colleagues, whereas Experiment 4 zoomed in on the largest and most diagnostic set size with a large number of trials to diagnose zero-information states in individual participants. In these two experiments, I was still not able to replicate the evidence that responses at later output positions were indistinguishable from guessing on the level of the entire sample, although a minority of participants showed behavior compatible with pure guessing.

## Method

### Participants

Students of the University of Zurich took part in each of the four experiments (*N* = 23, 24, 23, and 24 for Experiments 1, 2, 3, and 4, respectively) in exchange for partial course credit or monetary compensation consisting of either 45 Swiss Francs for three 1-hr sessions (Experiments 1–3) or 70 Swiss Francs for four 1-hr sessions (Experiment 4). The experiments were carried out in accordance with the guidelines of the Ethics Committee of the Faculty of Arts and Sciences at the University of Zurich.

### Materials and procedure

The materials and procedure were closely modeled after Experiment 1a by Adam et al. (2017), in which participants reproduced colors and were free to choose the order in which they reported all array items. The experiment was run with a program written in *MATLAB* (The MathWorks, Natick, MA) together with the *Psychophysics Toolbox 3* (Brainard, 1997), derived from the script by Adam et al.^1^

In Experiments 1 to 3, the set size of the arrays was 1, 2, 3, 4, or 6, with 90 trials per set size (30 in each session), presented in a random order. In Experiment 4, I used only Set Size 6—the most diagnostic for zero-information states—to maximize the number of trials per condition (*N* = 500, or 125 in each session). The stimuli were colored squares of 50 × 50 pixels, presented on a gray background. For each array, their colors were drawn at random with replacement from 360 colors in a color circle in Commission Internationale de l’Éclairage (CIE) Lab color space (centered at *L* = 54, *a* = 18, *b* = −8; Wyszecki & Stiles, 1982). The stimuli were placed at equidistant locations on an invisible circle (radius = 110 pixels) centered in the middle of the screen.

**Statement of Relevance**

When briefly presented with a rich scene, people can remember only a handful of visual objects from one glance to the next. This limitation reflects the capacity of visual working memory. The nature of this capacity limit has been a matter of intense debate. One view is that working memory has a discrete number of slots, each of which can hold one object. An alternative view is that all objects are held in working memory, though with variable degrees of strength or precision. Adam et al. (2017) provided an important piece of evidence for slot theories: When people report on the objects in a scene in a self-chosen order, they start with the objects they remember best. After reporting features of three to four objects, their responses become indistinguishable from guessing. The present experiments followed up on that finding and revealed little evidence for guessing-like response patterns, even for reports of up to six objects. Thus, the present results shift the balance of evidence substantially in favor of the alternative theories.

In Experiment 1, the stimuli were presented sequentially for 500 ms each, starting at the top of the invisible circle and proceeding in clockwise order. In Experiment 2, the stimuli were presented simultaneously for 250 ms multiplied by the set size. In Experiments 3 and 4, stimuli were presented simultaneously for 150 ms regardless of set size, as in the experiments by Adam et al.

After a 1-s retention interval during which the screen went blank, the color wheel was displayed in a new random rotation on each trial, together with dark-gray squares in the locations of the array items. Participants were instructed to report each item by choosing its color from the color wheel with a mouse click, and to do so in any order they liked. For each response, they had to first select the item to be reported by clicking on its place holder and then clicking on the color for that item. In this way, they had to report the colors of all items in the array. Participants were instructed to click on the colors they believed that they remembered—even if only vaguely—with the left mouse button, but when they did not know and were merely guessing, they were to click on them with the right mouse button.

To prevent participants from encoding the stimuli verbally in Experiments 1 and 2, I asked them to repeat aloud “ba bi bu” during presentation at a rate of one syllable every 500 ms (Experiment 1) or one syllable every 250 ms (Experiment 2). The speech rate was indicated by presenting the three syllables at the required rate on the screen before each trial. Participants’ speech was recorded and spot-checked for compliance.

No participants or data were excluded from analysis. Analyses were run in *JAGS* (Version 4.2.0; Plummer, 2016) and *R* (Version 3.6.2; R Core Team, 2019). All raw data and modeling code are available on OSF (https://osf.io/vwnbh/).

## Results

I first report results pertaining to the question of whether the data revealed evidence for zero-information states in VWM, followed by an exploratory analysis of people’s meta-cognitive accuracy. An analysis of the serial-position data from Experiment 1 can be found in the Supplemental Material available online.

### Evidence for memory states without information?

**Fig. 1.**
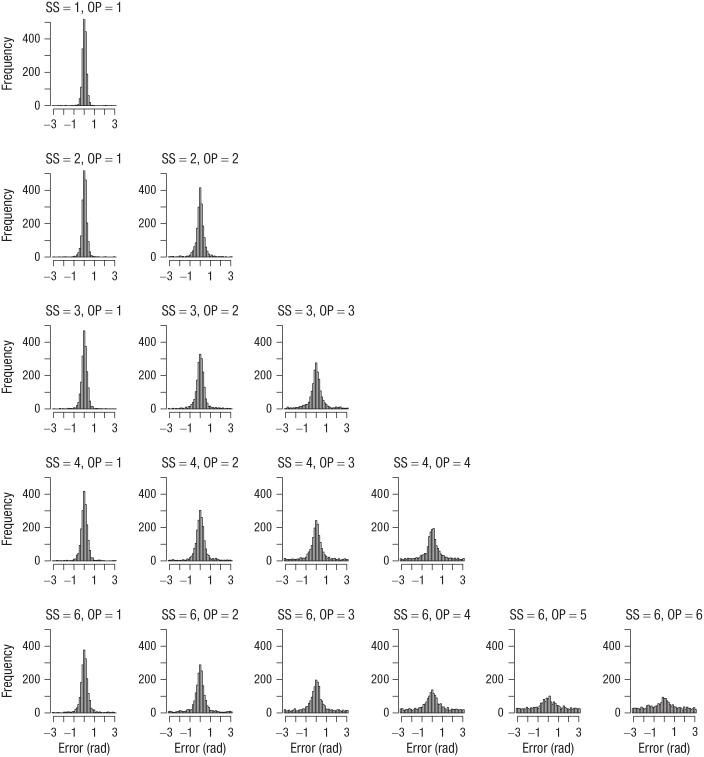
Error distributions for each combination of set size (SS) and output position (OP) in Experiment 1. Errors were computed as the angular deviation of the selected color from the true color in the color wheel.

**Fig. 2.**
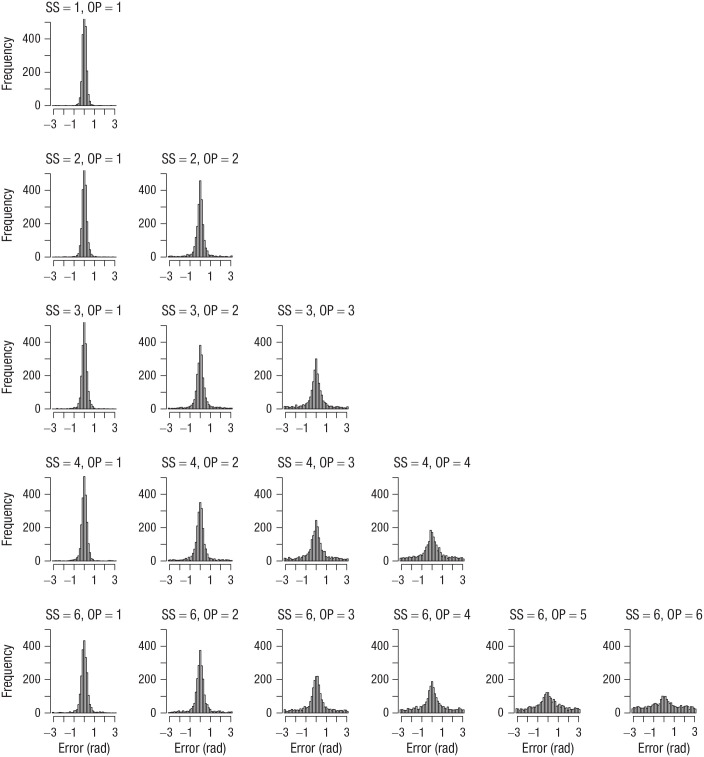
Error distributions for each combination of set size (SS) and output position (OP) in Experiment 2. Errors were computed as the angular deviation of the selected color from the true color in the color wheel.

**Fig. 3.**
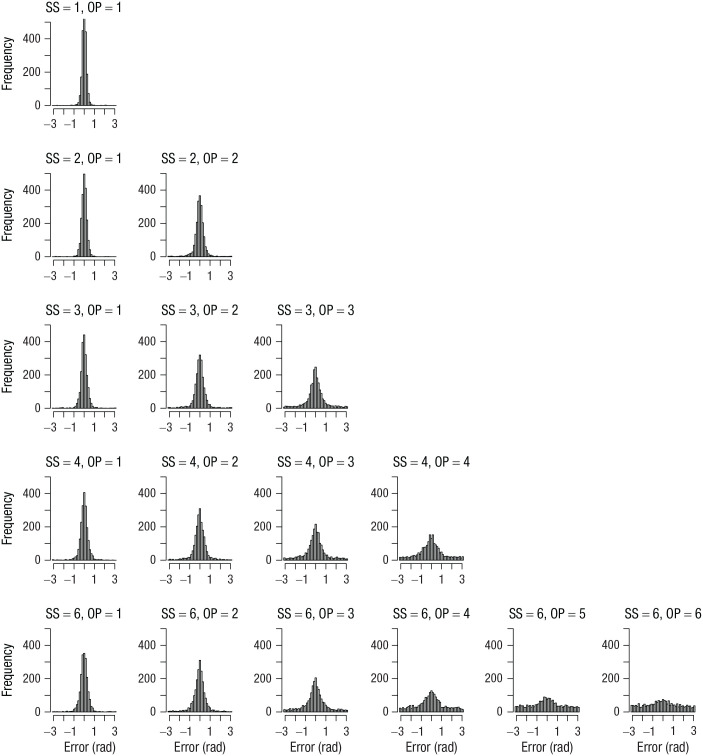
Error distributions for each combination of set size (SS) and output position (OP) in Experiment 3. Errors were computed as the angular deviation of the selected color from the true color in the color wheel.

**Fig. 4.**
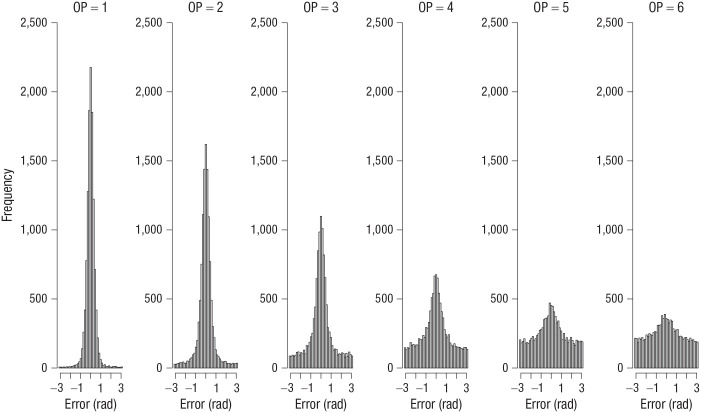
Error distributions for each output position (OP) in Experiment 4. Errors were computed as the angular deviation of the selected color from the true color in the color wheel.

I computed the error of each response as the absolute angular deviation of the selected color from the true color in the color wheel. Figure 1 shows the distribution of errors for each set size and output position from Experiment 1. Figures 2 to 4 show the analogous set of error distributions for Experiments 2 to 4, respectively. Whereas Adam et al. observed virtually flat (uniform) distributions of errors at output positions larger than three, this was not the case here: At every set size and output position, the error distributions peaked around zero, showing that participants had some information about the item’s color at least on a subset of trials.

To estimate the contribution of memory information to the error responses, I fitted a mixture model to the data of each experiment, separately for each set size and output position. The mixture model is a measurement model for continuous reproduction introduced by Zhang and Luck (2008) to measure the number of items for which people could, on average, give a response informed by memory of the target. The three-parameter version that I used was subsequently proposed by Bays et al. (2009). It describes the distribution of responses as a mixture of three distributions: (a) a von Mises distribution—a normal distribution on the circle—centered on the target item’s true color; (b) a set of von Mises distributions, each of which is centered on the color of one of the nontarget items in the array; and (c) a uniform distribution. The target-centered distribution (a) reflects memory of the to-be-reported item’s feature with a precision given by the dispersion parameter of the von Mises distribution. The nontarget centered distributions (b) reflect binding errors in VWM: People confuse the color of the item in the to-be-reported location with the color of another array item in another location, reflecting a failure of bindings between colors and their locations in memory. The uniform distribution (c) can be interpreted as reflecting guessing when people have no information about the to-be-reported item. The model has three free parameters: *P*(mem), the proportion of responses attributed to the target-centered distribution; *P*(nontarget), the proportion of responses attributed to nontargets, and κ, the precision of the von Mises distributions. The first two parameters jointly determine the proportion of responses attributed to guessing, *P*(uniform) = 1 – *P*(mem) – *P*(nontarget).

I used the Bayesian hierarchical implementation of the mixture model (Oberauer et al., 2017). The hierarchical model version describes each person’s parameter values as coming from a population distribution. Fitting the model yields parameter estimates for the population (i.e., mean and standard deviation of the population distributions) as well as for each person. Because the person parameter estimates are constrained by the population-level parameters, their estimates are informed not only by the data of that person but also—indirectly—by the data of all other participants, yielding more stable person parameter estimates than from models fitted to each individual separately. The Bayesian implementation yields posterior probability distributions for all parameters, which provide information not only about their location (e.g., the posterior mean or median) but also about the degree of certainty or uncertainty of the estimation (i.e., the dispersion of the posteriors). I applied the model with the same priors as in the study by Oberauer et al. (2017).

**Fig. 5.**
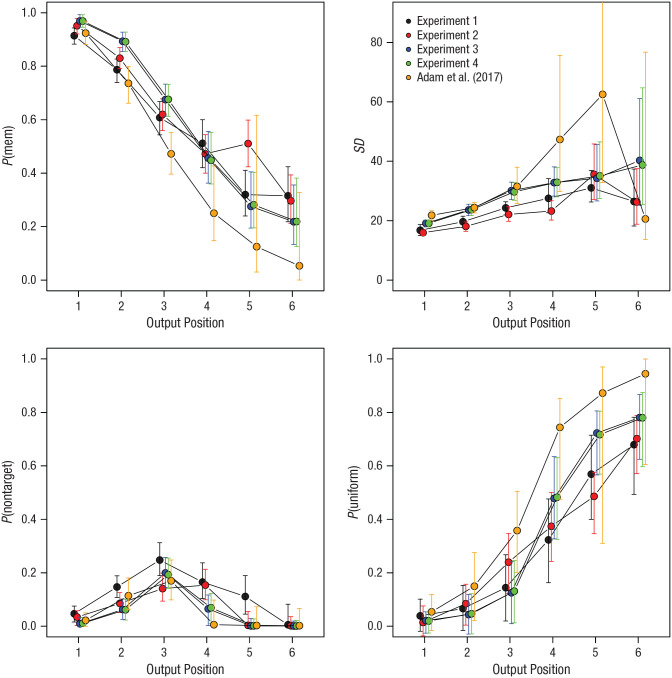
Estimates from the three-parameter mixture model for each output position of the Set Size 6 condition for five experiments: the four presented here and the original Experiment 1a by Adam et al. (2017). Each data point is the mode of the posterior distribution of the population-level mean parameter (the mode is the parameter value with the highest posterior probability). Error bars are 95% highest-density intervals.

Figure 5 shows the parameter estimates from the mixture model across output positions of Set Size 6 for five experiments: the four experiments presented here and the original Experiment 1a by Adam et al. Each data point is the mode of the posterior distribution of the population-level mean parameter (i.e., the parameter value with the highest posterior probability), and the error bars represent the 95% highest-density interval (i.e., the smallest interval in which the true parameter value lies with 95% posterior probability).

For the original experiment by Adam et al., the posterior of *P*(mem) dropped to values close to zero at the later output positions. Because the proportion of nontarget responses, after initially rising up to Output Position 3, also dropped to zero, this implies that the proportion of responses attributed to the uniform mixture component approached 1, as shown in the lower right panel of Figure 5. The highest-density interval of *P*(uniform) also reached 1, meaning that the assumption that all responses at the last one or two output positions come from a zero-information state is fairly credible. By contrast, for the present experiments, the posteriors of *P*(uniform) remained clearly below 1; their highest-density intervals all remained below .9. This means that the proportion of responses that can be attributed to zero-information states most likely does not exceed .9 at any output position. In other words, at every output position of the Set Size 6 condition of the present experiments, there were at least some trials that reflected information about the array rather than guessing.

### Are there individuals with zero-information states?

Whereas I found no evidence for zero-memory states in the population-level parameters, these parameters reflect only the average of the population. There remains the possibility that some individuals had zero-information states. I tried to identify such individuals through two converging approaches. First, I fitted the two-parameter mixture model (Zhang & Luck, 2008) to the data of each participant separately for all output positions of Set Sizes 4 and 6. I fitted the model twice to each data set—once with the Nelder-Mead algorithm as implemented in the *dfoptim* package (Version 2016-7-1; Varadhan & Borchers, 2020) and once with the limited-memory Broyden-Fletcher-Goldfarb-Shanno box constraints (L-BFGS-B) algorithm in the optim function of R—and kept the better of the two fits, that is, the one with the smaller deviance. (The deviance is equal to −2 × log likelihood.) For each participant and condition, I compared the fit of the mixture model with that of a uniform model that reflects 100% random guesses. For model comparison, I used the Bayesian information criterion (BIC), a fit indicator combining the deviance with a penalty for the extra flexibility that the mixture model has because of its two free parameters (the uniform model has none). When the BIC favored the uniform model, I assumed the participant had a zero-information state for the given set size and output position. Table 1 shows the number of participants (out of 24) in each experiment identified in this way as having a consistent zero-information state for a given design cell.

**Table 1.** Number of Participants for Whom a Uniform Model Fitted the Error Distributions Better Than a Two-Parameter Mixture Model, Separately for Each Combination of Set Size and Output Position (OP)

Experiment	Set size = 4				Set size = 6					
	OP = 1	OP = 2	OP = 3	OP = 4	OP = 1	OP = 2	OP = 3	OP = 4	OP = 5	OP = 6
1	0	0	0	0	0	0	0	2	9	10
2	0	0	0	0	0	0	0	1	5	10
3	0	0	0	0	0	0	0	3	9	15
4					0	0	1	3	6	11
Adam et al. (2017)	0	0	3	7	0	0	2	14	22	20

The data in Table 1 suggest that about half of the participants in each experiment had zero-information states for at least one combination of set size and output position. However, one needs to keep in mind that these results rest on model fits to the data of individual participants, which are more prone to distortion by measurement noise than the estimates from a hierarchical model.

To mitigate that limitation, I again used a Bayesian hierarchical modeling framework in the second approach. I extended the two-parameter mixture model (Zhang & Luck, 2008) by a second mixture parameter for the probability that a participant is in a consistent zero-information state for an entire condition of an experiment. This double-mixture model therefore has mixture parameters on two levels, the participant level and the trial level. On the participant level, the distribution of participants is modeled as a mixture of two populations: a population of participants who always respond in a guessing-like manner, producing uniformly distributed errors, and a population of participants who behave according to the Zhang and Luck mixture model. On the trial level, participants from the mixture-model population produce an error distribution that is a mixture of trials reflecting memory information and other trials reflecting guessing.

The hierarchical double-mixture model (HDMM) predicts each response *x̂_i,j_* of participant *j* in trial *i* as distributed according to a von Mises (VM) distribution:

$${\hat{x}}_{i,j}\sim \mathrm{VM}(m_{i,j};x_{j}z_{i,j}\kappa_{j})$$

The mean of the von Mises, *m_i,j_*, is the true feature of the target item. Conveniently, the von Mises distribution with a precision of 0 is a uniform distribution on the circle. Therefore, we can multiply the precision parameter κ_*j*_ with two binary variables, *x_j_* and *z_i,j_*, both drawn from a Bernoulli distribution:

$$x_{j}\sim \mathrm{Bernoulli}\left(P_{\mathrm{mix}}\right)$$

$$z_{i,j}\sim \mathrm{Bernoulli}\left(Pm_{j}\right)$$

The first equation, *x_j_*, expresses whether participant *j* is from the mixture-model population (*x_j_* = 1) or from the consistently guessing population (*x_j_* = 0). The probability that a participant comes from the mixture-model distribution is parameter *P*_mix_. The second binary variable, *z_i,j_*, expresses whether a mixture-model participant’s response on trial *i* actually comes from memory (*z_i,j_* = 1) or from guessing (*z_i,j_* = 0). The probability that person *j* responds on the basis of memory is given by parameter *Pm_j_*. If and only if both binary variables are 1, the response is modeled as coming from a von Mises distribution with precision κ; otherwise, it is drawn from a uniform distribution. The prior for *P*_mix_ was a uniform distribution in the range [0, 1].

The participant-level parameters are modeled as draws from a population distribution:

$$Pm_{j}\sim beta\left(A_{Pm},B_{Pm}\right)$$

$$\kappa_{j}\sim gamma(S_{\kappa },R_{\kappa })$$

The priors for these two distributions are the same as in the standard mixture model (Oberauer et al., 2017), with one exception: I used gamma(5, 0.2) as the prior for the mean of precision, which assigns low prior probability to small precision values. This represents the assumption that persons from the mixture-model population have a memory precision that is clearly distinguishable from guessing.

**Fig. 6.**
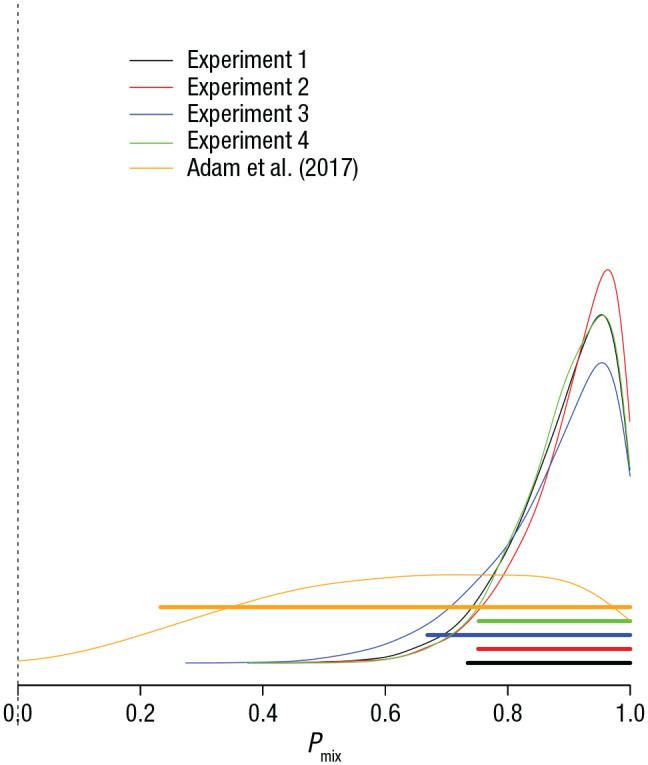
Posterior probability distribution of the *P_mix_* parameter from the double-mixture model, which reflects the probability that a participant comes from the hypothetical population of participants who have some memory information at Output Position 6 of the Set Size 6 condition. Results are shown separately for the four experiments presented here and the original Experiment 1a by Adam et al. (2017). Thick horizontal bars are 95% highest-density intervals of the posterior distributions.

I applied the HDMM to the data of the last output position of Set Size 6 in each experiment because that is the condition in which the first approach detected the most participants with uniform error distributions. Figure 6 shows the posterior distribution of *P*_mix_ for the five experiments. For the present experiments, high values of *P*_mix_ are most credible, implying that the probability of encountering a person with consistent guessing behavior in the population is low. For the data of Adam et al., by contrast, the posterior of *P*_mix_ was poorly located. This means that in light of the data, nearly every value of *P*_mix_ is credible. Hence, these data of Adam et al. are compatible with the conjecture that most or all participants are in consistent zero-information states, but they are equally compatible with the assumption that none of them are.

**Fig. 7.**
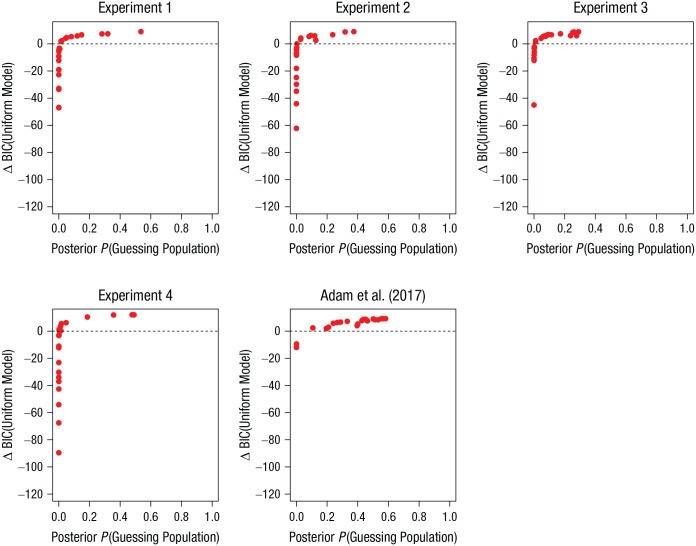
Correspondence of indicators of individual zero-information states: Bayesian information criterion (BIC) difference from the maximum-likelihood model comparison as a function of the posterior probability of a consistent zero-information state from the Bayesian hierarchical double-mixture model. Positive BIC differences reflect evidence for the uniform model over the two-parameter mixture model. Each data point comes from one participant in the Set Size 6, Output Position 6 condition. One data point with a BIC difference of −203 was omitted from Experiment 4.

For each participant, the proportion of posterior samples of *x_i_* that are zero provides an estimate of posterior probability that the participant belongs to the consistent-guessing population. Figure 7 plots the two indicators of individual zero-information states against each other—the BIC difference from the maximum-likelihood model comparison and the posterior probability of a consistent zero-information state from the Bayesian HDMM. The two indicators show a nearly perfect rank-order correlation in each experiment, but the posterior probability of *x_i_* = 0 from the HDMM rarely exceeded .5 even for participants for which the BIC difference was in favor of the uniform model over the mixture model.

**Fig. 8.**
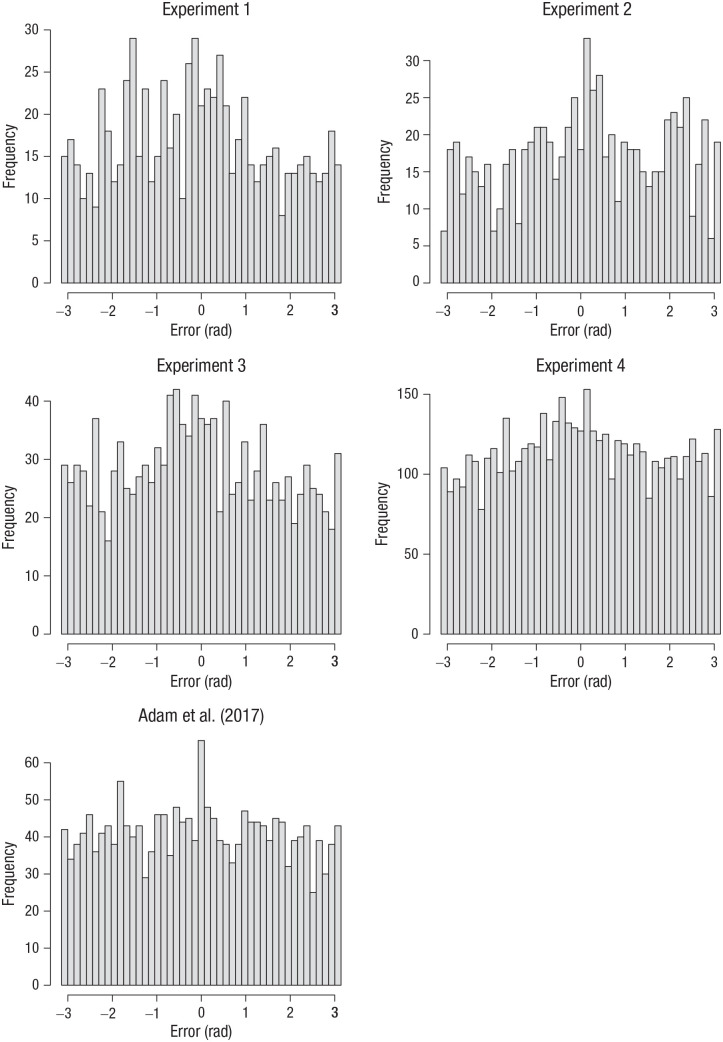
Error distributions from the Set Size 6, Output Position 6 condition for the subset of participants who were identified as having had a zero-information state by the individual maximum-likelihood fits of the two-parameter mixture model. Results are shown separately for the four experiments presented here and the original Experiment 1a by Adam et al. (2017). Errors were computed as the angular deviation of the selected color from the true color in the color wheel.

To further check the validity of the classification of error distributions as coming from zero-information states, I selected the data of Set Size 6, Output Position 6, from those participants whose data in that design cell were fitted better by a uniform model than the mixture model according to the BIC. Their error distributions are plotted in Figure 8. Whereas the error distribution of the data from Adam et al. plausibly came from a uniform distribution, those from the present experiments show a peak around zero, indicating that at least some of the participants identified as having had zero-information states had some memory for the true feature on at least some of the trials.

### Meta-cognitive accuracy

**Fig. 9.**
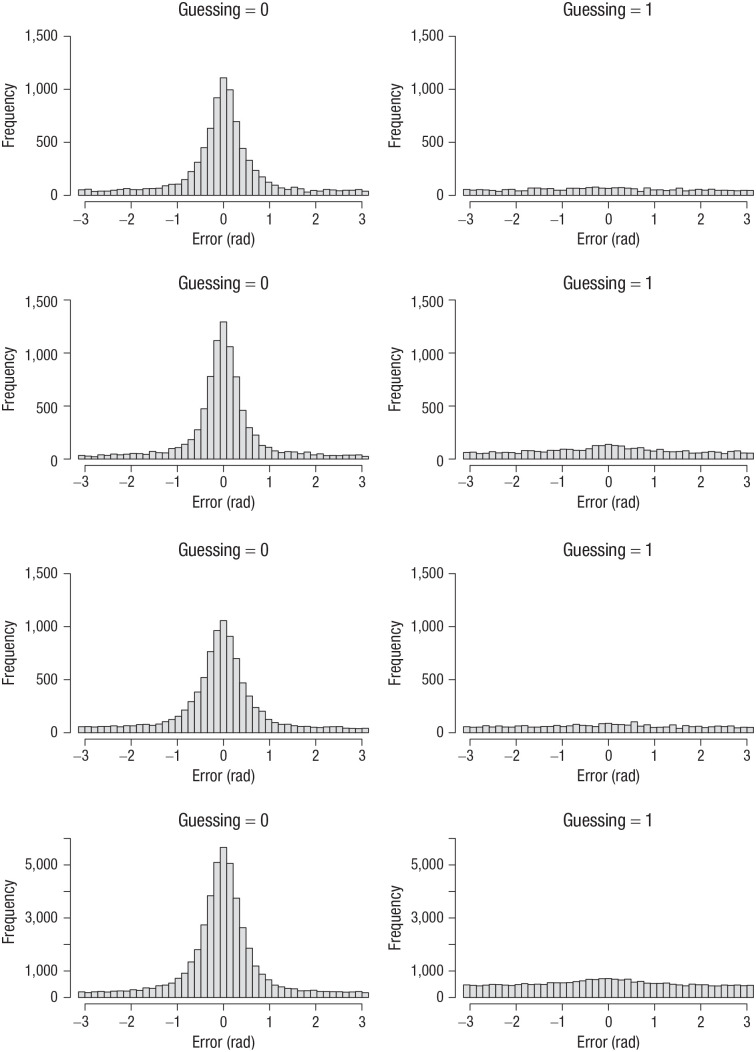
Error distributions from Set Size 6 for responses with the left mouse button (guessing = 0) and with the right mouse button (guessing = 1) in Experiments 1 to 4 (top row to bottom row, respectively). Errors were computed as the angular deviation of the selected color from the true color in the color wheel.

Participants were asked to respond with the left mouse button when they remembered the color and with the right mouse button when they thought they were only guessing. Figure 9 plots error distributions from Set Size 6—the condition with the most even split between self-reported memory-based and guessing responses. In all four experiments, the error distributions from self-reported guesses were approximately uniform.

Apparently, participants had good meta-cognitive insight into when their responses were informed by memory and when they were not—at least they were very good at identifying the latter situation. At the same time, the error distributions from self-reported memory-based responses still showed a substantial number of very large errors, reflected in the broad tails of the distributions. This could be because, as participants were instructed to use the right mouse button conservatively, they used the left mouse button in case of doubt. In this interpretation, the decision to use the left or the right mouse button would be made as a signal-detection process on a continuously varying degree of confidence, with a conservative criterion.

Alternatively, self-reported guesses could reflect a qualitatively different meta-cognitive state from self-reported memory-based responses. Self-reported guesses could reflect the trials on which participants made a deliberate decision to guess, perhaps because they found the attempt to remember the color hopeless or not worth the effort of a lengthy retrieval attempt. By contrast, responses with the left mouse button reflect genuine memory-based responses, some of which happen to reflect extremely poor, noisy memory, leading to very large errors. Future research measuring people’s confidence with a more fine-grained, approximately continuous scale could perhaps help to adjudicate between these possibilities.

People’s highly accurate meta-cognitive judgments agree with earlier findings showing a strong relation between confidence and precision of reproduction (Honig et al., 2020; Mitchell & Cusack, 2018; Rademaker et al., 2012). Moreover, they converge with the observation that the present participants—like those in the Adam et al. study—were very good at ordering their responses by reproduction accuracy.

## Discussion

My initial motivation in the present series of experiments was to investigate the possibility that the zero-information states that Adam et al. (2017) identified reflected limitations of encoding rather than limitations of working memory. The results provided evidence against that assumption: In Experiment 1, colors were presented sequentially at a relatively slow pace, giving participants ample opportunity to attend each of them. By contrast, Experiments 3 and 4 replicated the brief, simultaneous presentation of arrays as in the original study by Adam et al., which could have caused limitations of encoding. Neither overall performance nor the evidence for zero-information states in individual conditions or participants differed between these experiments. Therefore, presenting arrays of colors simultaneously for merely 150 ms does not impose a noticeable limit on encoding. This conclusion converges with those from previous studies that found only small, and inconsistent, differences between simultaneous and sequential presentation of simple visual stimuli (Gorgoraptis et al., 2011; Liu & Becker, 2013; Miller et al., 2014) and little gain in memory performance from exposure times longer than a few 100 ms (Bays et al., 2011; Vogel et al., 2006).

Nevertheless, none of the present experiments replicated the observation of uniformly distributed error distributions at the group level, which reflect essentially no information from memory. Therefore, the “clear evidence for item limits” that Adam et al. identified in their data was not found here. This result converges with the findings of a recent study by Bruning and Lewis-Peacock (2020), who tested continuous reproduction of six locations in a free-ordered whole-report procedure and found error distributions centered on the true location at all six output positions. An analysis on the level of participants revealed individual differences: About half of the participants in the present experiments showed evidence consistent with zero-information states and the other half did not; in the data of Adam and colleagues, only a small minority was identified as not showing zero-information states. Therefore, the present results do not deviate qualitatively from those of Adam and colleagues but quantitatively, reflecting a larger proportion of participants without zero-information states. Because the samples came from different student populations, it is plausible that they reflect a different mix of individual strategies. This heterogeneity underscores the limited generalizability of findings from convenience samples of students, such as the present one and those recruited by Adam et al.

Whereas the present findings do not lend support to the hypothesis of discrete item limits in VWM, they do not provide clear evidence against it either, for the following reason: The prediction of uniformly distributed error distributions at large set sizes and late output positions follows from the conjunction of two assumptions. One is that VWM is limited to a discrete number of items, and the other is that participants very consistently start reproducing the features of items they have in VWM, followed by guessing on those items that they do not have. If one of these assumptions is wrong, the prediction no longer holds. It is possible that the second assumption is wrong for the present experiments: Participants might have been less consistent in recalling the items they knew first. For instance, a person with an item limit of three or four could—at least on a substantial proportion of trials—give their report of a six-item array in a left-to-right, top-to-bottom order, regardless of which items they held in working memory. In this way, some responses even at the last output position would reflect memory, whereas some earlier responses would reflect guesses. To conclude, the design of the present experiments, and those of Adam et al., is suited only for providing evidence for the existence of zero-information states in VWM, not against it.

That said, the existence of uniform error distributions in the experiment by Adam et al. is not unambiguous evidence for discrete item limits either. The assumption of a discrete capacity limit entails that if set size exceeds capacity, people must have a zero-information state about some of the items, as reflected in a uniform error distribution on the circle. The converse, however, does not hold because there are alternative explanations for uniform error distributions. One is that they reflect the tail of a broad distribution of memory precisions (van den Berg et al., 2012): Precision fluctuates widely between and within trials, and some items are encoded with such low precision that their reproduction is not measurably better than guessing. A recent model comparison has shown a better quantitative account of the Adam et al. data by a variable-precision model than by a discrete-capacity model (Schneegans et al., 2020). This explanation would go along with the assumption that confidence varies continuously as a function of precision (van den Berg et al., 2017), and people make their decisions whether or not to report that they were guessing by placing a criterion on that continuum. Adam et al. argued that this is a theoretically unattractive proposition: A variable-precision model has to allow precision to vary so much that, in some conditions, the precision of items is indistinguishable from zero. Such a model would exploit the flexibility of a precision distribution that can be stretched out freely to mimic the behavior of a discrete-capacity model.

Another alternative explanation is that people have representations of nonnegligible precision of all array items but choose to guess on some responses nonetheless (Oberauer & Lin, 2017). Although doing so would mean sacrificing available memory information, it could still be rational because retrieving that information might not be worth the time investment when the information is difficult to retrieve and likely to be poor. In this account, there is a qualitative distinction between memory-based responses and guesses, but that distinction pertains not to the state of memory but to the person’s meta-cognitive decision on whether to use memory. Applied to the findings of Adam et al., this explanation is not very plausible because one has to assume that nearly every person decided to guess on nearly every trial at Output Position 4 and higher. Such a high agreement on a strategic decision that involves sacrificing some degree of response accuracy is unlikely. The present finding that, at best, about half of the participants produced response distributions indistinguishable from guessing substantially mitigates that problem: We only have to assume that some people decide to forfeit a small gain in accuracy by not retrieving weak memories. In light of individual differences in achievement motivation and in strategy choices, this is a fairly credible assumption.

To conclude, the existence of uniform error distributions is not decisive evidence for a discrete capacity limit on items in working memory, nor is their absence decisive evidence against it. Nevertheless, the demonstration of uniform error distributions in the experiments of Adam et al. precisely in those conditions in which one would expect them on the basis of a capacity limit to about three to four items increased the credibility of that hypothesis. Expressed in Bayesian terms, the likelihood of such a finding is high under the assumption of a discrete capacity limit and low—although not zero—for alternative models. It is lower because alternative models had to invoke auxiliary assumptions with low prior probability. Thereby, the discrete-capacity hypothesis gained in posterior probability relative to competing accounts of working memory capacity limitations. By the same rationale, the present findings shift the balance of evidence more in favor of the hypothesis that all items of a visual array are represented in VWM with some nonnegligible strength and precision, because the necessary assumptions in alternative models now have higher prior probability.

## Supplemental Material

sj-docx-1-pss-10.1177_09567976211068045 – Supplemental material for Little Support for Discrete Item Limits in Visual Working Memory Supplemental material, sj-docx-1-pss-10.1177_09567976211068045 for Little Support for Discrete Item Limits in Visual Working Memory by Klaus Oberauer in Psychological Science

## Transparency

*Action Editor:* Barbara Knowlton

*Editor:* Patricia J. Bauer

**Open Practices:** All raw data and modeling code have been made publicly available via OSF and can be accessed at https://osf.io/vwnbh/. The design and analysis plan for the experiments were not preregistered.

## Notes

1. I am grateful to Kirsten Adam, Ed Vogel, and Ed Awh for sharing their MATLAB script and their raw data on OSF.
