# Supplementary material for: Little Support for Discrete Item Limits in Visual Working Memory
Source: Psychol Sci. 2022 Jun 17;33(7):1128–42. doi: 10.1177/09567976211068045 (PMC13038119; doi:10.1177/09567976211068045)
Supplement: sj-docx-1-pss-10.1177_09567976211068045 – Supplemental material for Little Support for Discrete Item Limits in Visual Working Memory [file sj-docx-1-pss-10.1177_09567976211068045.docx]

**Supplementary Material**

**Serial-Position Effects**

Experiment 1, in which items were presented sequentially, offers the opportunity to analyze reproduction errors and self-reported guesses as a function of the items’ serial position in the presentation sequence (i.e., input position), as well as their position in the recall sequence (i.e., output position). We can also investigate how people’s self-chosen order of recall depends on the order of presentation.

Participants showed a strong tendency to report the items in the order in which they were presented, and that tendency was strongest for the smaller set sizes. Table S1 shows the frequencies of the 7 most popular output orders at each set size

*Table S1: Frequencies of the 7 most popular output orders in Experiment 1*

| Set Size 2  Order | Frequency | Set Size 3 Order | Frequency | Set Size 4 Order | Frequency | Set Size 6 Order | Frequency |
| --- | --- | --- | --- | --- | --- | --- | --- |
| 12 | 1829 | 123 | 1421 | 1234 | 919 | 123456 | 486 |
| 21 | 119 | 132 | 221 | 1423 | 145 | 612345 | 124 |
|  |  | 321 | 122 | 1324 | 130 | 654321 | 11 |
|  |  | 312 | 75 | 1243 | 107 | 162345 | 68 |
|  |  | 231 | 63 | 4321 | 99 | 561234 | 61 |
|  |  | 213 | 46 | 4123 | 92 | 651234 | 51 |
|  |  |  |  | 1432 | 69 | 165432 | 40 |

*Note: Orders are given by listing the input positions of reported items in the order in which they were reported.*

This observation matches findings from free-recall tasks with words, and with spatial locations (Cortis, Dent, Kennett, & Ward, 2015; Grenfell-Essam, Ward, & Tan, 2013): For list lengths up to about 6 items, people have a preference for starting recall with the first-presented item, and tend to proceed in forward order. For that reason, the following analyses are split by trials in which participants’ output order strictly matched the presentation order, and trials in which it did not.

Figure S1 shows the mean absolute error (on the left) and the proportion of trials on which participants indicated that they were merely guessing by responding with the right mouse button (right) as a function of input position (= output position) for those trials that were reported in strict forward order. Errors show the serial-position curves typical for forward serial recall of verbal and spatial lists (for a review see Oberauer et al., 2018), with an extended primacy effect and a small recency effect, observed here only for the largest set size. A similar pattern has been found with serial continuous-reproduction of colors (Peteranderl & Oberauer, 2018). The proportion of self-reported guesses increased steadily across output positions, but they remained a minority of trials even at the last output position.


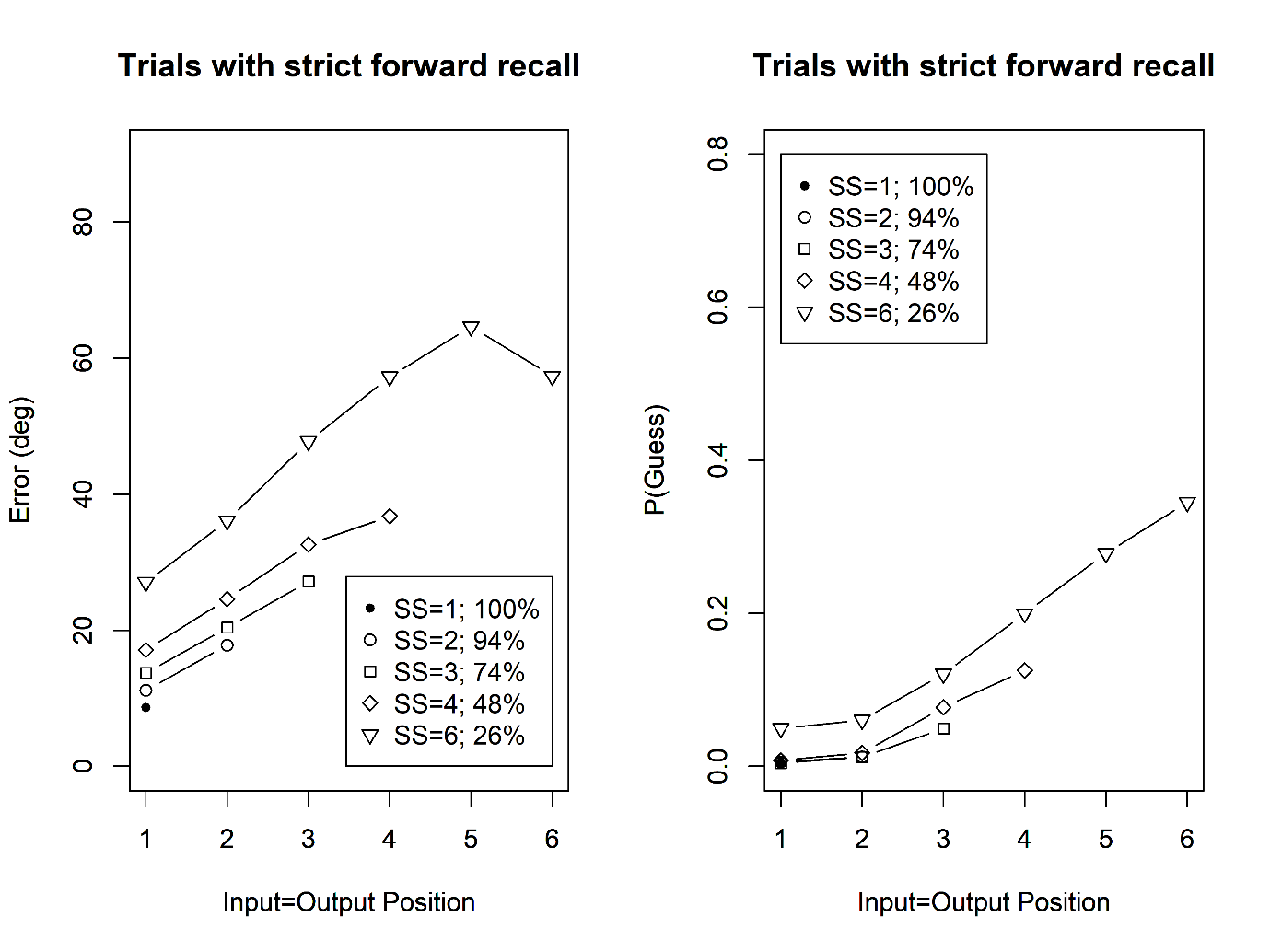


*Figure S1: Mean errors (absolute deviation of response from target color in degrees) and proportion of self-reported guessing responses in Experiment 1 for the subset of trials in which the colors were reported strictly in their order of presentation. SS = set size; the percentage in the legend is the percentage of trials reported in strict forward order.*

The corresponding serial-position curves for the trials not recalled in forward order are plotted in Figure S2 as a function of input order in the top panels, and as a function of output order in the bottom panels. The serial-position curve of errors mirror those observed with verbal materials when items are probed in a random order to disentangle input and output position effects (Oberauer, 2003): There were approximately symmetric primacy and recency effects over input position, and a steady decline of performance across output positions. The same trends can be seen in the proportions of self-reported guesses.


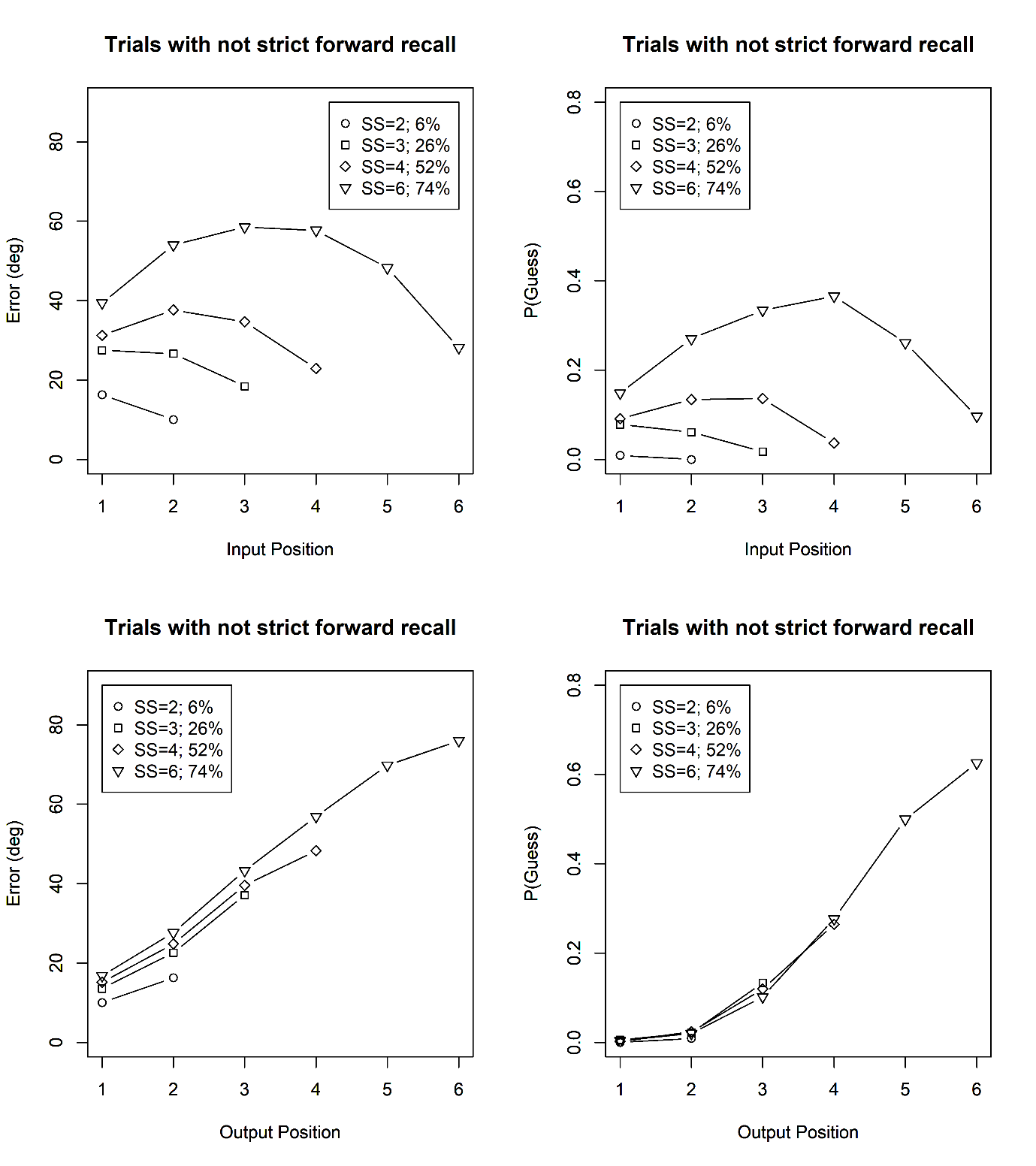


*Figure S2: Mean errors (absolute deviation of response from target color in degrees) and proportion of self-reported guessing responses in Experiment 1 for the subset of trials in which the colors were not reported in their order of presentation. SS = set size; the percentage in the legend is the percentage of trials reported in strict forward order.*

Compared to the trials reported in forward order, here the proportion of self-reported guesses was substantially higher, whereas performance was at a comparable level. This could be a hint that people chose to recall in forward order to the extent that they were confident to be able to recall all items – in agreement with the observation that people’s inclination to recall a list in forward order decreases with larger set size, and also when recall is made harder by other experimental manipulations (Grenfell-Essam et al., 2013).

Taken together, the effects of serial position on reproduction accuracy mirror findings from list-memory studies using other (primarily verbal) materials, supporting the assumption that primacy and recency effects, and the tendency to recall short lists in forward order in free-recall tests, reflect general principles of memory.
